# Classification of 5-S Epileptic EEG Recordings Using Distribution Entropy and Sample Entropy

**DOI:** 10.3389/fphys.2016.00136

**Published:** 2016-04-14

**Authors:** Peng Li, Chandan Karmakar, Chang Yan, Marimuthu Palaniswami, Changchun Liu

**Affiliations:** ^1^School of Control Science and Engineering, Shandong UniversityJinan, China; ^2^Centre of Pattern Recognition and Data Analytics (PRaDA), Deakin UniversityGeelong, VIC, Australia; ^3^Electrical and Electronic Engineering Department, University of MelbourneMelbourne, VIC, Australia

**Keywords:** electroencephalogram (EEG), epileptic seizure, distribution entropy (DistEn), sample entropy (SampEn), short-length EEG analysis

## Abstract

Epilepsy is an electrophysiological disorder of the brain, the hallmark of which is recurrent and unprovoked seizures. Electroencephalogram (EEG) measures electrical activity of the brain that is commonly applied as a non-invasive technique for seizure detection. Although a vast number of publications have been published on intelligent algorithms to classify interictal and ictal EEG, it remains an open question whether they can be detected using short-length EEG recordings. In this study, we proposed three protocols to select 5 s EEG segment for classifying interictal and ictal EEG from normal. We used the publicly-accessible Bonn database, which consists of normal, interical, and ictal EEG signals with a length of 4097 sampling points (23.6 s) per record. In this study, we selected three segments of 868 points (5 s) length from each recordings and evaluated results for each of them separately. The well-studied irregularity measure—sample entropy (SampEn)—and a more recently proposed complexity measure—distribution entropy (DistEn)—were used as classification features. A total of 20 combinations of input parameters *m* and τ for the calculation of SampEn and DistEn were selected for compatibility. Results showed that SampEn was undefined for half of the used combinations of input parameters and indicated a large intra-class variance. Moreover, DistEn performed robustly for short-length EEG data indicating relative independence from input parameters and small intra-class fluctuations. In addition, it showed acceptable performance for all three classification problems (interictal EEG from normal, ictal EEG from normal, and ictal EEG from interictal) compared to SampEn, which showed better results only for distinguishing normal EEG from interictal and ictal. Both SampEn and DistEn showed good reproducibility and consistency, as evidenced by the independence of results on analysing protocol.

## Introduction

Epilepsy is the fourth most common neurological disorder after migraine, stroke, and Alzheimer's disease (Sirven and Shafer, 2014) with an estimated 50 million people globally living with epilepsy (Media-Center, 2015). Epilepsy occurs in people of all ages and can affect them economically, socially, and even culturally. People with epilepsy often experience reduced educational opportunities, barriers to particular occupations, reduced access to health and life insurance, and other social stigma and discrimination (Sirven and Shafer, 2014; Media-Center, 2015). Recent studies show that up to 70% of people with epilepsy can be successfully treated. However, about three fourths in low- and middle-income countries may not receive the treatment they need. This is a considerable “treatment gap,” since nearly 80% of the epilepsy population live in those countries (Media-Center, 2015). Barriers to treatment for those people include the lack of trained healthcare providers and reliable low-cost diagnostic techniques (Media-Center, 2015).

Some common reasons of epilepsy are an abnormality in brain connections, an increased synchronization of neuronal activity in the brain (in which some brain cells either over-excite or over-inhibit other cells), a brain damage associated with conditions that disrupt normal brain activity, or some combination of these factors (NINDS, 2015). The hallmark of epilepsy is recurrent and unprovoked seizures. During the “epileptogenesis” process, the normal neuronal network abruptly turns into a hyper-excitable network, affecting mostly the cerebral cortex (Acharya et al., 2013). The most commonly used diagnostic tests for epilepsy is the measurement of electrical activity in the brain through monitoring electroencephalogram (EEG) or magnetoencephalogram (MEG) signals, and brain scans including computed tomography (CT), positron emission tomography (PET), and magnetic resonance imaging (MRI) (NINDS, 2015; Zhang et al., 2015).

EEG is a non-invasive, low-cost, yet effective technique for examining the electrical activity of the brain. Abnormal spike discharges can be identified in EEG recordings before and during a seizure attack (interictal and ictal states). Recognition of interical and ictal seizure phases through the analysis of EEG features has long been studied (Acharya et al., 2013). Those EEG features are selected from a wide spectrum, including time-domain (Meier et al., 2008; Minasyan et al., 2010), frequency-domain (Polat and Güneş, 2007; Chua et al., 2011), time-frequency analysis (Ocak, 2009; Tzallas et al., 2009; Guo et al., 2010; Alam and Bhuiyan, 2013; Kumar et al., 2014a,b), and features based on non-linear dynamics of the signal (Yuan et al., 2011). Non-linear methods have attracted increasing attention recently, since EEG signals are considered outputs of an intrinsically non-linear, complex system—the brain. Published studies have explored the availability of different non-linear methods, especially entropy features such as approximate entropy (ApEn) (Srinivasan et al., 2007; Ocak, 2009; Guo et al., 2010; Kumar et al., 2014a,b), sample entropy (SampEn) (Song et al., 2012), fuzzy entropy (FuzzyEn) (Kumar et al., 2014a; Xiang et al., 2015), and permutation entropy (PE) (Nicolaou and Georgiou, 2012; Li et al., 2014), or the combinations of two or more of these entropy features (Kannathal et al., 2005; Yuan et al., 2011; Acharya et al., 2012, 2015), all of which have shown good performance for distinguishing interictal, ictal EEG signals, and normal signals.

However, these studies are based on the entire EEG recordings (whose lengths is usually more than 20 s) from a specific database (Srinivasan et al., 2007; Ocak, 2009; Guo et al., 2010; Yuan et al., 2011; Acharya et al., 2012; Nicolaou and Georgiou, 2012; Song et al., 2012; Kumar et al., 2014a,b). The reason for using longer data length may partly be due to the fact that the traditional entropy methods are parameter dependent and typically can achieve stable estimations only for relatively long data recordings (e.g., 1000 sampling points or more; Richman and Moorman, 2000; Xie et al., 2008; Chen et al., 2009; Yentes et al., 2013). Therefore, most of the existing algorithms are only suitable for offline or post event detection of seizure rather than online or during event detection. This limits the caregivers to take prompt action during an event, which is important for better health outcome of epileptic patient. Patients may be exposed to life-threatening conditions if a seizure onset cannot be detected promptly. Additionally, online epilepsy and seizure detection based on short-length EEG recordings is set to become increasingly favored with the emergence of portable EEG amplifiers. To the best of our knowledge, there is currently no published study that has systematically attempted to achieve accurate detection using short-length EEG recordings.

In 2015, Li et al. (2015a) developed a new entropy method—distribution entropy (DistEn)—based on the distribution of inter-vector distances in the state space representation of time-series. DistEn has shown superiority for analysis of both benchmark data (Li et al., 2015a; Udhayakumar et al., 2015) and real world clinical data (Li et al., 2015) with extremely small number of samples compared with SampEn and FuzzyEn. In addition, DistEn precludes the dependence upon input parameters of the traditional methods (Li et al., 2015a; Udhayakumar et al., 2015). In our previous study, we applied this novel DistEn method to analyzing normal, interictal, and ictal EEGs, and found significant differences between the interical and ictal EEGs (Li et al., 2015b). Additionally, in that study we have explored how the length of EEGs influences DistEn performance. We tested the between group differences of DistEn using complete recording (4097 samples), average of each 5 s (868 samples) and 1 s (174 samples) epochs over complete recording, respectively. Intriguingly, we found that the performance of average DistEn of 5 s epochs was almost the same as that was found using the complete EEG recording. On the other hand, when using 1 s epochs, DistEn was not only able to detect the difference between interical and ictal EEGs, but also the difference between normal and interictal EEGs. Although the study used 5 and 1 s epoch, it is not true case of short length application, since it was averaged over the complete recording. Therefore, in the current study we will use only one epoch instead. We have decided to use epoch length of 5 s rather than 1 s in this study as we believe that 1 s epoch is too short and other algorithms (such as SampEn) mostly cannot give in valid results.

One important aspect of using a short-length segments from long recording is the selection process of the segment of interest from the recording. Most studies follow the random selection procedure, which presents difficulties with regard to evaluating the reproducibility and generalizability of the technique. Since the choice of data segment mostly affects the feature and hence the overall results, the reliability of the findings becomes questionable. To address this problem, we proposed three segmentation protocols and evaluated results for each of them separately (Li et al., 2015b).

**Figure 1:**
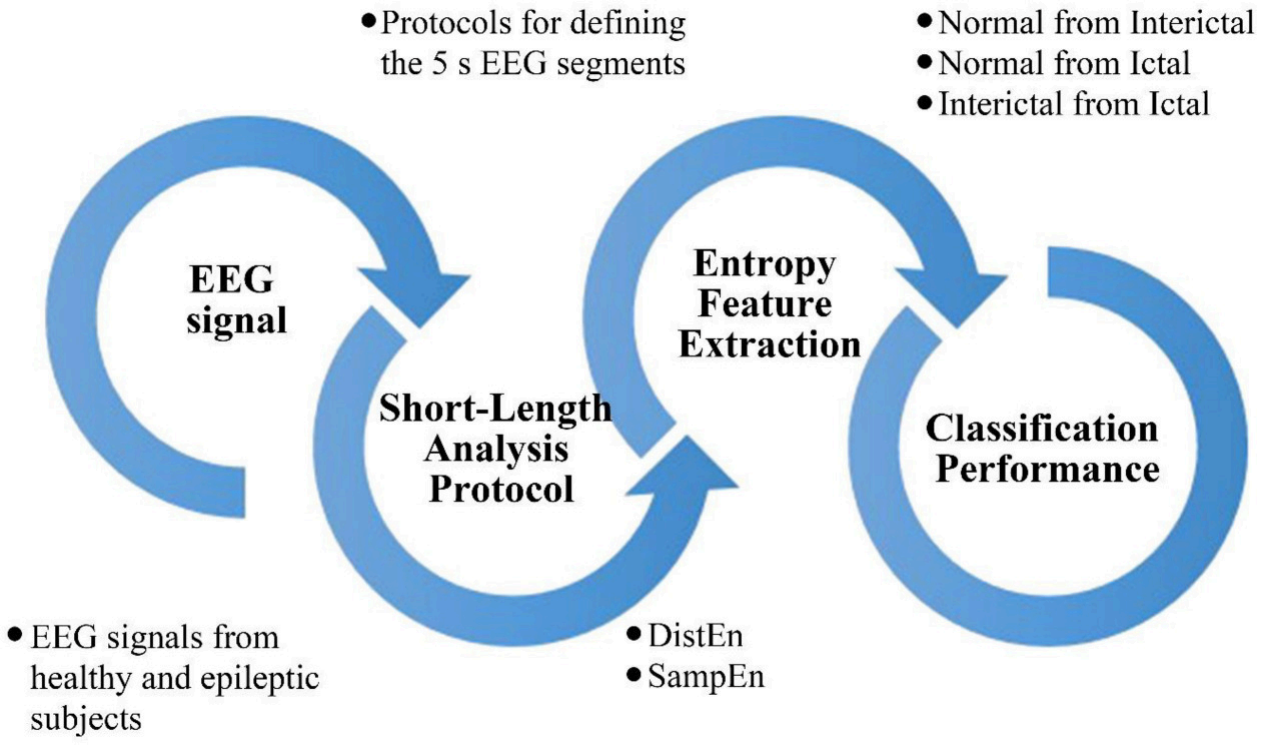
**Overall block diagram summarizing steps of this study**.

In this study, we compared the performance of the DistEn and SampEn methods for classifying short-length epileptic EEG recordings with a data length of 5 s. Figure 1 shows a block diagram of this study. At first, we collected EEG signals from healthy and epileptic subjects from online database and proposed three protocols for the selection of 5 s EEG signal from complete recording. Then we used the DistEn and SampEn for feature extraction. Finally, we evaluated and compared the classification performance of extracted features among Normal, Interictal, and Ictal groups.

Algorithms of DistEn and SampEn are described in Section Algorithms of DistEn and SampEn. Section Description of EEG Data summarizes the EEG data used in this study. Statistical analysis methods are provided in Section Statistical Analysis. Results are provided in Section Results, followed by discussions in Section Discussion. Conclusions are presented in the last Section.

## Materials and methods

### Algorithms of DistEn and SampEn

#### DistEn

DistEn measures the complexity of a time-series through quantifying the amount of information contained in the inter-vector distances of the state space representation of the time-series. Through the evaluation of benchmark data, it has been demonstrated that a time-series with chaotic regime will result in dispersedly distributed inter-vector distances, leading to a larger amount of information, whereas the distribution becomes concentrative for random and periodic time-series, leading to relatively smaller amount of information (Li et al., 2015a). The algorithm for determining DistEn value of a time-series of *N* samples {*u*(*i*), 1 ≤ *i* ≤ *N*} can be summarized as follows:

- State space reconstruction: Form (*N* − (*m* − 1)τ) vectors **X**(*i*) by **X**(*i*) = {*u*(*i*), *u*(*i* + τ), …, *u*(*i* + (*m* − 1)τ)}, 1 ≤ *i* ≤ *N* − (*m* − 1)τ. Here *m* indicates the embedding dimension and τ the time delay.
- Distance matrix construction: Compute the inter-vector distances (distances between all possible combinations of **X**(*i*) and **X**(*j*)) by *d*_*i, j*_ = max(|*u*(*i* + *k*) − *u*(*j* + *k*)|, 0 ≤ *k* ≤ *m* − 1) for all 1 ≤ *i, j* ≤ *N* − *m*. The distance matrix is denoted as **D** = {*d*_*i, j*_}.
- Probability density estimation: Estimate the empirical probability density function of the distance matrix **D** by the histogram approach with a fixed bin number of *B*. The probability of each bin can be denoted as {*p*_*t*_, *t* = 1, 2, …, *B*}. Note here elements with *i* = *j* in **D** are excluded in the estimation.
- Calculation: The DistEn value of the time-series {*u*(*i*)} can be calculated by the formula for Shannon entropy, that is
  (1) $$\begin{array}{r} \text{DistEn}\left(m,\;\tau ,B\right)=-\frac{1}{\log_{2}\left(B\right)}\sum_{t=1}^{B}p_{t}\log_{2}\left(p_{t}\right) \end{array}$$

#### SampEn

SampEn provides an estimation of time-series complexity via the quantification of its self-similarity or regularity. By definition, SampEn is the negative natural logarithm of the conditional probability that two vectors (in the state space representation) similar for *m* points will remain similar at the next point (Richman and Moorman, 2000). For a random time-series, similar vectors of observations will not likely be followed by additional similar observations, thus yielding a higher SampEn. On the contrary, a periodic time-series will have a relatively small SampEn because it contains many repetitive patterns. The following algorithm can be used to determine the SampEn value of a time-series of *N* points {*u*(*i*), 1 ≤ *i* ≤ *N*}.

- State space reconstruction: Form (*N* − *mτ*) vectors **X**(*i*) by **X**(*i*) = {*u*(*i*), *u*(*i* + τ), …, *u*(*i* + (*m* − 1)τ)}, 1 ≤ *i* ≤ *N* − *mτ*. Here *m* indicates the embedding dimension and τ the time delay.
- Ranking similar vectors: Define the distance between **X**(*i*) and **X**(*j*) (1 ≤ *i, j* ≤ *N* − *mτ, i* ≠ *j*) by *d*_*i, j*_ = max(|*u*(*i* + *k*) − *u*(*j* + *k*)|, 0 ≤ *k* ≤ *m* − 1). Denote $A_{i}^{\left(m\right)}\left(r\right)$ the average number of vectors **X**(*j*) within *r* of **X**(*i*) (that means *d*_*i, j*_ ≤ *r*) for all *j* = 1, 2, …, *N* − *mτ* and *j* ≠ *i* to exclude self-matches. Similarly, we define $A_{i}^{\left(m+1\right)}\left(r\right)$ to rank the similarity between vectors with next point added in the comparison. Here *r* indicates the threshold parameter.
- Calculation: The SampEn value of the time-series {*u*(*i*)} can be calculated by
  (2) $$\begin{array}{l} \text{SampEn}\;(m,\;\tau ,\;r)=-ln\frac{\sum_{i\;=\;1}^{N-m\tau }A_{i}^{\left(m+1\right)}\left(r\right)}{\sum_{i\;=\;1}^{N-m\tau }A_{i}^{\left(m\right)}\left(r\right)} \end{array}$$

#### Selection of input parameters

DistEn is a function of input parameters *m*, τ, and *B*, and SampEn a function of *m*, τ, and *r*, as described in the Sections DistEn and SampEn. According to Li et al. (2015a), DistEn is minimally affected by the assignment for *B*. In this study, we set *B* = 64 for all DistEn calculations. We used this value for *B* mainly due to the fact that we are analyzing short-length time-series and a small *B*-value is adequate for approximating the distribution of the inter-vector distances (Li et al., 2015a). On the other hand, the threshold parameter *r* is, indeed, a crucial factor for SampEn, since different *r*-values often lead to large variation in SampEn results (Richman and Moorman, 2000). This parameter-dependence may be further aggravated for a short data set (Yentes et al., 2013). In this study we are not exploring the effect of *r* on SampEn and therefore set *r* = 0.15*SD* (*SD* indicates the standard deviation of the time-series under consideration) empirically (Richman and Moorman, 2000; Yentes et al., 2013).

The embedding dimension *m* and time delay τ are important parameters since they together determine whether the state space reconstruction is appropriate or not. Several methods exist for determining the optimal values for *m* and τ either separately (Fraser and Swinney, 1986; Kennel et al., 1992) or jointly (Gautama et al., 2003). We used a differential entropy based method to determine the optimal *m* and τ jointly in order to avoid them falling into a non-optimal range (Gautama et al., 2003). Our analysis resulted in an optimal range of [2, 5] for *m* and [8, 12] for τ. In this study, we used all possible combinations of those *m*-and τ-values, yielding a total of 20 DistEn/SampEn values for each EEG segment. Details on the determination of *m* and τ are provided in the Supplementary Material.

### Description of EEG data

The EEG data used in this study come from the Bonn database (Andrzejak et al., 2001b). The database is publicly available online (Andrzejak et al., 2001a) and has been widely used in epilepsy and seizure detection research. It is a collection of a total of 500 single-channel EEG recordings of 23.6 s duration each. They are categorized into five groups (sets Z, O, N, F, and S) of 100 recordings each. Sets Z and O consist of surface EEG recordings collected from five healthy volunteers in awake and relaxed state, with their eyes open and closed, respectively, using the standard 10–20 electrode placement scheme. Sets N, F, and S are recorded from five epileptic patients through intracranial electrodes for interictal and ictal activities. Signals in set F are recorded from the epileptogenic zone during seizure-free intervals (interictal activities). Set N also contains only interictal EEG signals, which are recorded from the hippocampal formation of the opposite hemisphere of the brain. Set S contains only signals corresponding to seizure attacks (ictal activities). In this study, we devided the data sets in three groups: (i) Normal–Z, O; (ii) Interictal–N, F; and (iii) Ictal–S.

**Figure 2:**
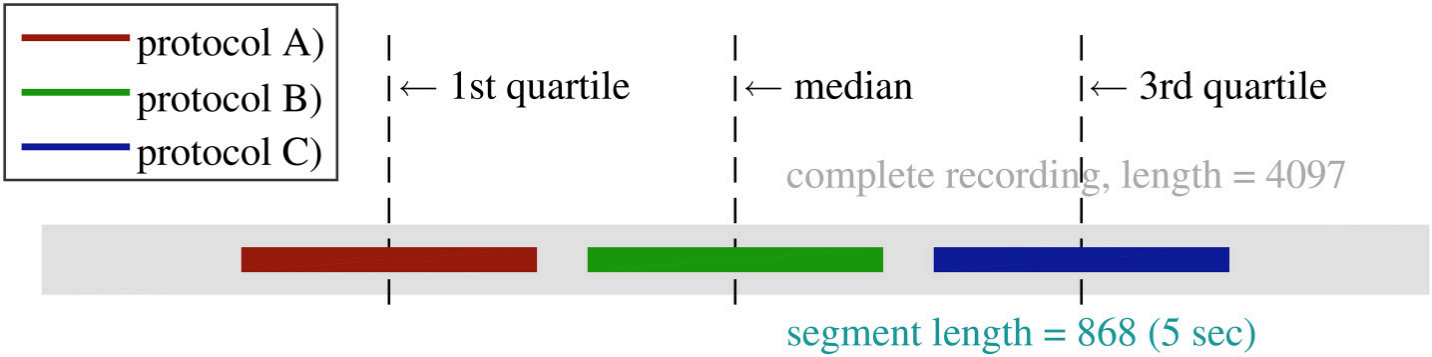
**Schematic diagram of the three 5 s segment selection protocols**.

All EEG recordings are digitized at 173.61 samples per second. Thus, the length of each recording is 173.61 × 23.6 ≈ 4097 samples, which is adequate for achieving robust estimations of entropy. However, in this study we concentrated on short-length EEG with duration of 5 s (173.61 × 5 ≈ 868). In order to select 5 s segment from each EEG recording (23.6 s), we proposed three protocols: (A) The 5 s segment was taken from the earlier stage of recording with its center aligned at the first quartile; (B) It was taken from the middle stage with its center aligned at the median; (C) The segment was taken from the later stage of recording with its center aligned at the third quartile. Figure 2 shows a schematic diagram of the three protocols.

### Statistical analysis

Statistical analysis include: (i) Mann-Whitney *U*−test to determine the differences of DistEn and SampEn, respectively, among normal (sets Z and O), interictal (sets N and F), and ictal EEGs as well as differences between each pair; (ii) ROC analysis to determine the classification performance of the two entropy methods for detecting interictal EEG from normal, ictal EEG from normal, and ictal from interictal EEG. All statistical analysis was performed individually for results obtained from each analysing protocol. Statistical significance was accepted at *p* < 0.01. Statistical analysis was performed using the Matlab R2014b (MathWorks, Natick, Massachusetts, USA).

The Mann-Whitney *U*-test was employed because the entropy results generally follow a non-normal distribution. *p* < 0.001 was accepted for statistical significance for parsimony in multiple comparisons. In ROC analysis, the classification performance was evaluated in term of area under the curve (AUC).

## Results

### DistEn and SampEn results for different EEG groups

Table 1 summarizes results of the 20 DistEn indices for normal, interictal, and ictal EEG groups and Table 2 the SampEn indices, each incorporating results corresponding to all the three analyzing protocols.

**Table 1:** **DistEn of normal, interictal, and ictal EEG recordings**.

**Feature Index**	**A**			**B**			**C**		
	**Normal**	**Interictal**	**Ictal**	**Normal**	**Interictal**	**Ictal**	**Normal**	**Interictal**	**Ictal**
1^†,†,†^	0.85 ± 0.03	0.86 ± 0.03^c^	0.89 ± 0.05^b^	0.85 ± 0.03^a^	0.86 ± 0.03^c^	0.90 ± 0.03^b^	0.85 ± 0.03	0.86 ± 0.04^c^	0.89 ± 0.05^b^
2^†,†,†^	0.85 ± 0.03^a^	0.86 ± 0.03^c^	0.89 ± 0.05^b^	0.85 ± 0.03^a^	0.86 ± 0.03^c^	0.90 ± 0.03^b^	0.85 ± 0.03	0.86 ± 0.03^c^	0.89 ± 0.05^b^
3^†,†,†^	0.85 ± 0.03^a^	0.86 ± 0.03^c^	0.89 ± 0.05^b^	0.85 ± 0.03^a^	0.86 ± 0.03^c^	0.89 ± 0.03^b^	0.85 ± 0.03	0.86 ± 0.03^c^	0.89 ± 0.05^b^
4^†,†,†^	0.85 ± 0.03^a^	0.86 ± 0.03^c^	0.90 ± 0.05^b^	0.85 ± 0.03^a^	0.86 ± 0.03^c^	0.89 ± 0.03^b^	0.85 ± 0.03	0.86 ± 0.03^c^	0.88 ± 0.05^b^
5^†,†,†^	0.85 ± 0.03^a^	0.86 ± 0.03^c^	0.90 ± 0.05^b^	0.85 ± 0.03^a^	0.86 ± 0.03^c^	0.89 ± 0.03^b^	0.85 ± 0.03	0.86 ± 0.03^c^	0.88 ± 0.05^b^
6^†,†,†^	0.85 ± 0.02^a^	0.86 ± 0.03^c^	0.90 ± 0.04^b^	0.85 ± 0.03^a^	0.86 ± 0.03^c^	0.90 ± 0.04^b^	0.85 ± 0.03^a^	0.86 ± 0.03^c^	0.89 ± 0.05^b^
7^†,†,†^	0.85 ± 0.03^a^	0.86 ± 0.03^c^	0.90 ± 0.04^b^	0.85 ± 0.02^a^	0.86 ± 0.03^c^	0.90 ± 0.04^b^	0.85 ± 0.03^a^	0.86 ± 0.03^c^	0.89 ± 0.05^b^
8^†,†,†^	0.85 ± 0.02^a^	0.86 ± 0.03^c^	0.90 ± 0.04^b^	0.85 ± 0.03^a^	0.86 ± 0.03^c^	0.90 ± 0.04^b^	0.85 ± 0.02^a^	0.86 ± 0.03^c^	0.89 ± 0.05^b^
9^†,†,†^	0.85 ± 0.02^a^	0.86 ± 0.03^c^	0.90 ± 0.04^b^	0.85 ± 0.03^a^	0.86 ± 0.03^c^	0.90 ± 0.04^b^	0.85 ± 0.02^a^	0.86 ± 0.03^c^	0.89 ± 0.05^b^
10^†,†,†^	0.85 ± 0.02^a^	0.87 ± 0.03^c^	0.90 ± 0.04^b^	0.85 ± 0.03^a^	0.86 ± 0.03^c^	0.90 ± 0.04^b^	0.85 ± 0.02^a^	0.86 ± 0.03^c^	0.89 ± 0.05^b^
11^†,†,†^	0.85 ± 0.02^a^	0.87 ± 0.03^c^	0.90 ± 0.04^b^	0.84 ± 0.02^a^	0.86 ± 0.03^c^	0.90 ± 0.04^b^	0.85 ± 0.02^a^	0.86 ± 0.03^c^	0.89 ± 0.05^b^
12^†,†,†^	0.85 ± 0.02^a^	0.87 ± 0.03^c^	0.90 ± 0.04^b^	0.84 ± 0.02^a^	0.86 ± 0.03^c^	0.90 ± 0.04^b^	0.85 ± 0.03^a^	0.86 ± 0.03^c^	0.89 ± 0.05^b^
13^†,†,†^	0.85 ± 0.02^a^	0.87 ± 0.03^c^	0.90 ± 0.04^b^	0.84 ± 0.02^a^	0.86 ± 0.02^c^	0.90 ± 0.04^b^	0.85 ± 0.02^a^	0.86 ± 0.03^c^	0.89 ± 0.05^b^
14^†,†,†^	0.85 ± 0.02^a^	0.87 ± 0.03^c^	0.90 ± 0.04^b^	0.84 ± 0.02^a^	0.86 ± 0.03^c^	0.90 ± 0.04^b^	0.85 ± 0.02^a^	0.86 ± 0.03^c^	0.89 ± 0.05^b^
15^†,†,†^	0.85 ± 0.02^a^	0.87 ± 0.03^c^	0.90 ± 0.04^b^	0.84 ± 0.02^a^	0.86 ± 0.03^c^	0.90 ± 0.04^b^	0.85 ± 0.03^a^	0.86 ± 0.03^c^	0.89 ± 0.05^b^
16^†,†,†^	0.84 ± 0.02^a^	0.87 ± 0.03^c^	0.89 ± 0.04^b^	0.84 ± 0.02^a^	0.86 ± 0.02^c^	0.89 ± 0.04^b^	0.84 ± 0.02^a^	0.86 ± 0.03^c^	0.89 ± 0.05^b^
17^†,†,†^	0.84 ± 0.02^a^	0.87 ± 0.03^c^	0.89 ± 0.04^b^	0.84 ± 0.02^a^	0.86 ± 0.02^c^	0.89 ± 0.04^b^	0.84 ± 0.02^a^	0.86 ± 0.03^c^	0.89 ± 0.05^b^
18^†,†,†^	0.84 ± 0.02^a^	0.86 ± 0.03^c^	0.89 ± 0.04^b^	0.84 ± 0.02^a^	0.86 ± 0.02^c^	0.89 ± 0.04^b^	0.84 ± 0.02^a^	0.86 ± 0.03^c^	0.89 ± 0.05^b^
19^†,†,†^	0.84 ± 0.02^a^	0.86 ± 0.03^c^	0.89 ± 0.04^b^	0.84 ± 0.02^a^	0.86 ± 0.02^c^	0.89 ± 0.04^b^	0.84 ± 0.02^a^	0.86 ± 0.03^c^	0.88 ± 0.05^b^
20^†,†,†^	0.84 ± 0.02^a^	0.86 ± 0.03^c^	0.89 ± 0.04^b^	0.84 ± 0.02^a^	0.86 ± 0.02^c^	0.89 ± 0.04^b^	0.84 ± 0.02^a^	0.86 ± 0.03^c^	0.88 ± 0.05^b^

Values are given in Median ± IQR (inter quartile range). Results corresponding to different analyzing protocols are listed in columns A, B, and C, respectively.

^†^*: p < 0.001.*

a Interictal significantly different (p < 0.001) from Normal.

b Ictal significantly different (p < 0.001) from Normal.

c Ictal significantly different (p < 0.001) from Interictal. The p-values are indicated by Mann–Whitney U-test. Strings next to feature index indicate the significant levels for all three analyzing protocols, e.g., the string

†,†,† indicates p < 0.001 for protocols A, B, and C. Feature index 1, 2, … , 20 indicate the [m, τ] combinations [2, 8], [2, 9], … .,[5, 12], respectively.

**Table 2:** **SampEn of normal, interictal, and ictal EEG recordings**.

**Feature Index**	**A**			**B**			**C**		
	**Normal**	**Interictal**	**Ictal**	**Normal**	**Interictal**	**Ictal**	**Normal**	**Interictal**	**Ictal**
1^†,†,†^	2.32 ± 0.18^a^	1.92 ± 0.38	1.67 ± 0.49^b^	2.29 ± 0.23^a^	1.89 ± 0.39	1.76 ± 0.44^b^	2.29 ± 0.21^a^	1.89 ± 0.41	1.84 ± 0.58^b^
2^†,†,†^	2.34 ± 0.21^a^	1.92 ± 0.38	1.72 ± 0.50^b^	2.31 ± 0.18^a^	1.90 ± 0.40	1.81 ± 0.48^b^	2.30 ± 0.16^a^	1.90 ± 0.47	1.83 ± 0.53^b^
3^†,†,†^	2.35 ± 0.17^a^	1.94 ± 0.37	1.78 ± 0.47^b^	2.31 ± 0.15^a^	1.93 ± 0.38	1.82 ± 0.44^b^	2.32 ± 0.15^a^	1.94 ± 0.42	1.84 ± 0.46^b^
4^†,†,†^	2.34 ± 0.16^a^	1.93 ± 0.37	1.81 ± 0.42^b^	2.31 ± 0.16^a^	1.91 ± 0.38	1.87 ± 0.41^b^	2.32 ± 0.15^a^	1.95 ± 0.44	1.89 ± 0.47^b^
5^†,†,†^	2.33 ± 0.17^a^	1.92 ± 0.34	1.83 ± 0.41^b^	2.32 ± 0.17^a^	1.92 ± 0.37	1.88 ± 0.39^b^	2.31 ± 0.17^a^	1.93 ± 0.39	1.93 ± 0.48^b^
6^†,†,†^	2.16 ± 0.38^a^	1.38 ± 0.50	1.43 ± 0.53^b^	2.11 ± 0.38^a^	1.33 ± 0.51	1.46 ± 0.42^b^	2.10 ± 0.32^a^	1.36 ± 0.54	1.50 ± 0.63^b^
7^†,†,†^	2.17 ± 0.38^a^	1.35 ± 0.49	1.46 ± 0.53^b^	2.11 ± 0.34^a^	1.30 ± 0.48	1.45 ± 0.46^b^	2.14 ± 0.36^a^	1.38 ± 0.59	1.55 ± 0.58^b^
8^†,†,†^	2.15 ± 0.35^a^	1.33 ± 0.48	1.50 ± 0.48^b^	2.16 ± 0.34^a^	1.32 ± 0.55	1.54 ± 0.43^b^	2.13 ± 0.35^a^	1.38 ± 0.52	1.52 ± 0.57^b^
9^†,†,†^	2.20 ± 0.34^a^	1.29 ± 0.48	1.54 ± 0.51^b^	2.12 ± 0.34^a^	1.32 ± 0.45	1.56 ± 0.42^b^	2.12 ± 0.34^a^	1.34 ± 0.52	1.56 ± 0.61^b^
10^†,†,†^	2.18 ± 0.35^a^	1.31 ± 0.52	1.55 ± 0.54^b^	2.16 ± 0.34^a^	1.29 ± 0.47	1.57 ± 0.35^b^	2.18 ± 0.33^a^	1.34 ± 0.57	1.60 ± 0.57^b^
11~20	Undefined	Undefined	Undefined	Undefined	Undefined	Undefined	Undefined	Undefined	Undefined

Values are given in Median ± IQR (inter quartile range). Results corresponding to different analyzing protocols are listed in columns A, B, and C, respectively.

^†^ p < 0.001.

a Interictal significantly different (p < 0.001) from Normal.

b Ictal significantly different (p < 0.001) from Normal.

^c^Ictal significantly different (p < 0.001) from Interictal. The p-values are indicated by Mann–Whitney U-test. Strings next to feature index indicate the significant levels for all three analyzing protocols, e.g., the string

†,†,† indicates p < 0.001 for protocols A, B, and C. Feature index 1, 2, … , 20 indicate the [m, τ] combinations [2, 8], [2, 9], … ., [5, 12], respectively.

Significant differences among the three classes (normal, interictal, and ictal) are indicated by all the 20 DistEn indices (all *p* < 0.001). In contrast, only the first 10 SampEn indices (that means, SampEn with combinations of [*m*, τ] = [2, 8], [2, 9], [2, 10], [2, 11], [2, 12], [3, 8], [3, 9], [3, 10], [3, 11], and [3, 12]) show significant differences. The SampEn indices for the rest of the combinations of [*m*, τ] are undefined. More importantly, SampEn indices remained undefined for all three segmentation protocols (Table 2) and both DistEn and SampEn showed similar results over three protocols as indicated by Tables 1, 2.

Additionally, all the 20 DistEn indices show significant differences between each pair of classes except the first one DistEn index for protocol A and the first five indices for protocol C. Significant differences between normal and interictal EEGs, as well as between normal and ictal EEGs, are shown by the first 10 SampEn indices for all the three protocols. However, difference between interictal and ictal classes indicated by SampEn indices are statistically insignificant.

The Median values of DistEn are almost unchanged in all three groups (Normal, Interictal, and Ictal) over three 5 s segmentation protocols (A, B, and C) at all combinations of [*m*, τ] (Table 1). On the other hand, SampEn shows relative higher fluctuations in Median values across three protocols than DistEn (Tables 1, 2). Besides, the inter quartile range (IQR) values of DistEn are also obviously smaller than SampEn.

### ROC analysis results

**Figure 3:**
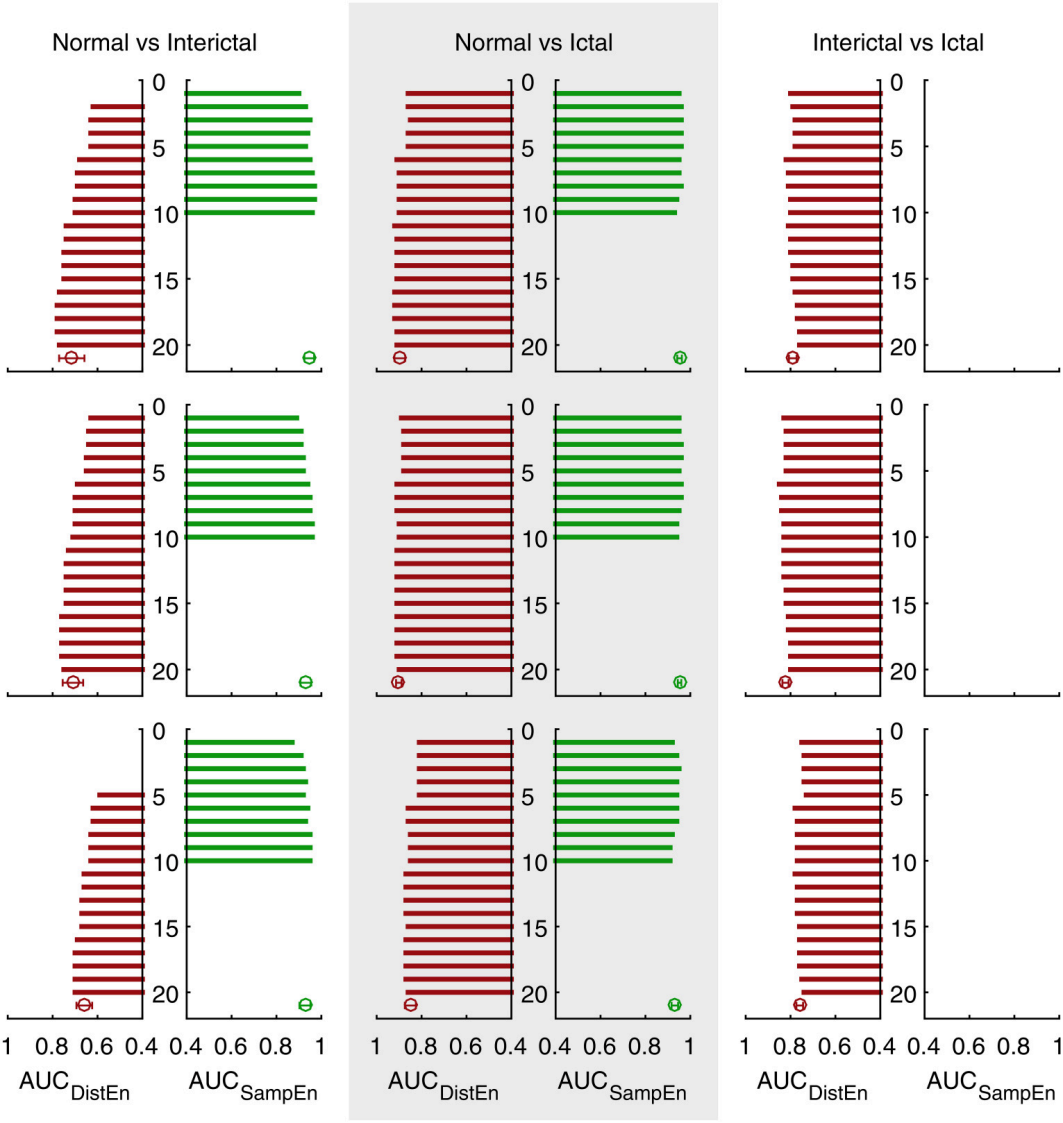
**Area under the ROC curves (AUC) of three different protocols (see Supplementary Figures 1–3 for detailed ROC results)**. Bars show AUC of ROC curves with feature indices from 1 to 20. Error bars show the average and standard deviation of the corresponding AUC sets (undefined values excluded).

ROC curves for DistEn and SampEn indices that have significant difference between classes (see Tables 1, 2) under different protocols are shown in Supplementary Figures 1, 3, respectively. Figure 3 summarizes the AUC values for all those ROC curves.

DistEn indices achieve an average AUC of 0.71 and maximum of 0.78 for detecting interictal EEG from normal (protocol A). Although SampEn indices are not always defined, they achieve an average AUC of 0.95 and maximum of 0.97 when only those valid features are taken into consideration. For detecting ictal EEG from normal, DistEn indices achieve an average AUC of 0.90 and maximum of 0.92 whereas SampEn of 0.95 on average and 0.96 for maximum (only valid SampEn indices are taken into consideration). Finally, for detecting ictal EEG from interictal, DistEn indices achieve an average AUC of 0.80 and maximum of 0.82; SampEn fails in this classification task because all SampEn indices show no significant difference between these two classes (see Table 2). No remarkable difference is found when comparing results obtained under different protocols (see Table 3).

**Table 3:** **Average and maximum Area under the ROC curves (AUC) values of DistEn and SampEn for different classification problems under different analyzing protocols**.

**Protocol**	**Method**	**Normal vs. Interictal**		**Normal vs. Ictal**		**Interictal vs. Ictal**	
		**Average**	**Maximum**	**Average**	**Maximum**	**Average**	**Maximum**
A	DistEn	0.71	0.78	0.90	0.92	0.80	0.82
	SampEn	0.95	**0.97**	0.95	**0.96**	-	-
B	DistEn	0.71	0.76	0.90	0.91	0.82	**0.85**
	SampEn	0.93	0.96	0.95	**0.96**	-	-
C	DistEn	0.66	0.70	0.85	0.87	0.76	0.78
	SampEn	0.93	0.95	0.93	0.95	-	-

*Bold indicates the maximum AUC for each of the three classification problems*.

## Discussion

In this work, we used the DistEn and SampEn methods to analyze short-length, specifically 5 s, EEG recordings with the aim of detecting interictal and ictal EEG timely and accurately. Although both methods showed the capability of differentiating one or more classes from others, differences in their performance were indicated. Besides, we found that results from one entropy method mostly complement the other:

- DistEn showed acceptable performance for all the three classification problems with high AUC values (see Figure 3). SampEn failed to distinguish ictal EEG from interictal, but it has shown good performance on the other two tasks. Furthermore, for these two problems SampEn was superior to DistEn as evidenced by a demonstrable increase in AUC values.
- SampEn was not always defined. For a half of our used combinations of input *m* and τ, SampEn frequently yielded invalid results (see Table 2). Whenever defined, SampEn has showed extremely high AUC values for detecting interictal EEG from normal and detecting ictal EEG for optimal combinations of [*m*, τ]. Thus, it is important to define the optimal range of *m* and τ in order to use SampEn as classification feature.
- DistEn was shown to be independent of input parameters *m* and τ, as can be seen from the relatively unchanged median and considerably smaller IQR values with the variation of *m* and τ (see Table 1). This property of DistEn can greatly facilitate clinical practices since the selection of input parameters is usually highly intractable.
- With optimal *m* and τ parameters, we can obtain an AUC of 0.97 for detecting interictal EEG from normal, 0.96 for detecting ictal EEG from normal, and 0.85 for detecting ictal EEG from interictal (see Table 3). Our results indicate that accurate detection of interictal and ictal seizure phases using short-length EEG recordings is possible by combining DistEn and SampEn analysis.

In this study we used three 5 s EEG segment selection protocols, which were non-overlapping. However, the performance of both DistEn and SampEn did not show remarkable differences with different protocols (see Tables 1, 2, Figure 3, and Table 3, respectively). This indicates a good reproducibility and consistency for these methods.

DistEn showed significant differences between normal and ictal EEGs as well as between interictal and ictal EEGs which is in accordance with our previous findings based on averaging 5 s epochs (Li et al., 2015b), suggesting that the use of a 5 s epoch is reasonable. Intriguingly, DistEn also demonstrated acceptable performance for detecting interictal EEG from normal, which cannot be achieved by the previous analysis (Li et al., 2015b). Since in that study we used the default selection of *m* and τ (*m* = 2, τ = 1), the results reported here could well underline the importance of searching optimal input parameters. This is also another noticeable difference of our study compared with some previous publications (Srinivasan et al., 2007; Ocak, 2009; Guo et al., 2010; Yuan et al., 2011; Acharya et al., 2012, 2015; Song et al., 2012; Yentes et al., 2013; Kumar et al., 2014a,b) additional to short-length analysis. Although DistEn has shown rather stable outputs with the variation of [*m*, τ], SampEn did change considerably and even with some combinations of [*m*, τ] SampEn could not yield valid results as we have mentioned above. Besides, Yuan et al. (2008) have demonstrated that the embedding dimension of EEG signals during seizure changes and becomes different from that of normal EEG signals; the embedding dimension varies intensively during seizure, whereas keeps stable for normal EEG signals. Thus, applying a constant embedding dimension *m* may not be proper. By searching for optimal values and using different [*m*, τ] combinations, our methods may also have a good generalization capability because the sampling frequency varies much for different EEG databases or clinical EEG data which will affect the reconstruction in state space and is important deterministic factor to define *m* and τ (Thuraisingham and Gottwald, 2006; Govindan et al., 2007). Methods of some previous publications based on SampEn (Acharya et al., 2012, 2015; Song et al., 2012; Shen et al., 2013; Xiang et al., 2015), although having indicated good performance, may deserve further discussions when transplanted to other EEG data.

The brain exhibits randomness in normal state and changes to deterministic dynamics during ictal state. Our results indicated decreased SampEn and increased DistEn during both interictal and ictal EEG data relative to normal state. Since SampEn increases with the randomness or irregularity of a time-series (Richman and Moorman, 2000; Costa et al., 2005), higher SampEn in normal EEG data can be attributed to the stochastic dynamics of EEG signals. On the other hand, DistEn is reported to increase in non-linear deterministic dynamics (Li et al., 2015a) and thus, the increased DistEn in epileptic EEG signals can be attributed to the shift to deterministic dynamics in seizure activity. Therefore, although DistEn and SampEn have shown different variation directions, they represent two different characteristics of the signal.

One limitation of our study is that we did not try different values for the threshold parameter *r* for SampEn analysis. There may be an *r*-value that can support the capability of SampEn to detect ictal EEG from interictal. However, in clinical practice, it is impossible to change the *r*-values since it makes the results incomparable. Another limitation is that we did not test the generalization capability of our method, although actually it is expected to be good as we have mentioned above, by applying on other EEG databases.

## Conclusion

Interical and ictal phases of epileptic seizure can be detected using short-length EEG data of 5 s length. The SampEn method is more sensitive to the detection of epileptic EEG (including interical and ictal phases) from normal whereas the DistEn method is sensitive to not only the detection of epileptic EEG from normal but also the detection of ictal EEG from interictal. Through SampEn analysis a maximum AUC value of 0.97 was achieved for detecting interictal EEG from normal, and of 0.96 for detecting ictal EEG from normal. A maximum AUC of 0.85 was achieved by DistEn analysis for detecting ictal EEG from interictal. The results of this study have shown that real time detection of epileptic seizure is possible using portable EEG amplifiers.

## Author contributions

PL, CK, MP, and CL contributed to the conceptualization of the study. PL, CK, and CY analyzed the data and interpreted the results. PL drafted the manuscript. CK and PL critically revised the significant intellectual content of the work. All authors approved the final version of the manuscript.

### Conflict of interest statement

The authors declare that the research was conducted in the absence of any commercial or financial relationships that could be construed as a potential conflict of interest.
