# Supplementary material for: Classification of 5-S Epileptic EEG Recordings Using Distribution Entropy and Sample Entropy
Source: Front Physiol. 2016 Apr 14;7:136. doi: 10.3389/fphys.2016.00136 (PMC4830849; doi:10.3389/fphys.2016.00136)
Supplement: Supplementary file 1 [file DataSheet1.DOCX]

Supplementary Material

Classification of Five-Second Epileptic EEG Recordings Using Distribution Entropy and Sample Entropy

Peng Li^*^, Chandan Karmakar^*^, Chang Yan, Marimuthu Palaniswami, Changchun Liu

*** Correspondence:** Peng Li: [pli@sdu.edu.cn](mailto:pli@sdu.edu.cn)

Chandan Karmakar: [karmakar@deakin.edu.au](mailto:karmakar@deakin.edu.au)

# Supplementary Results


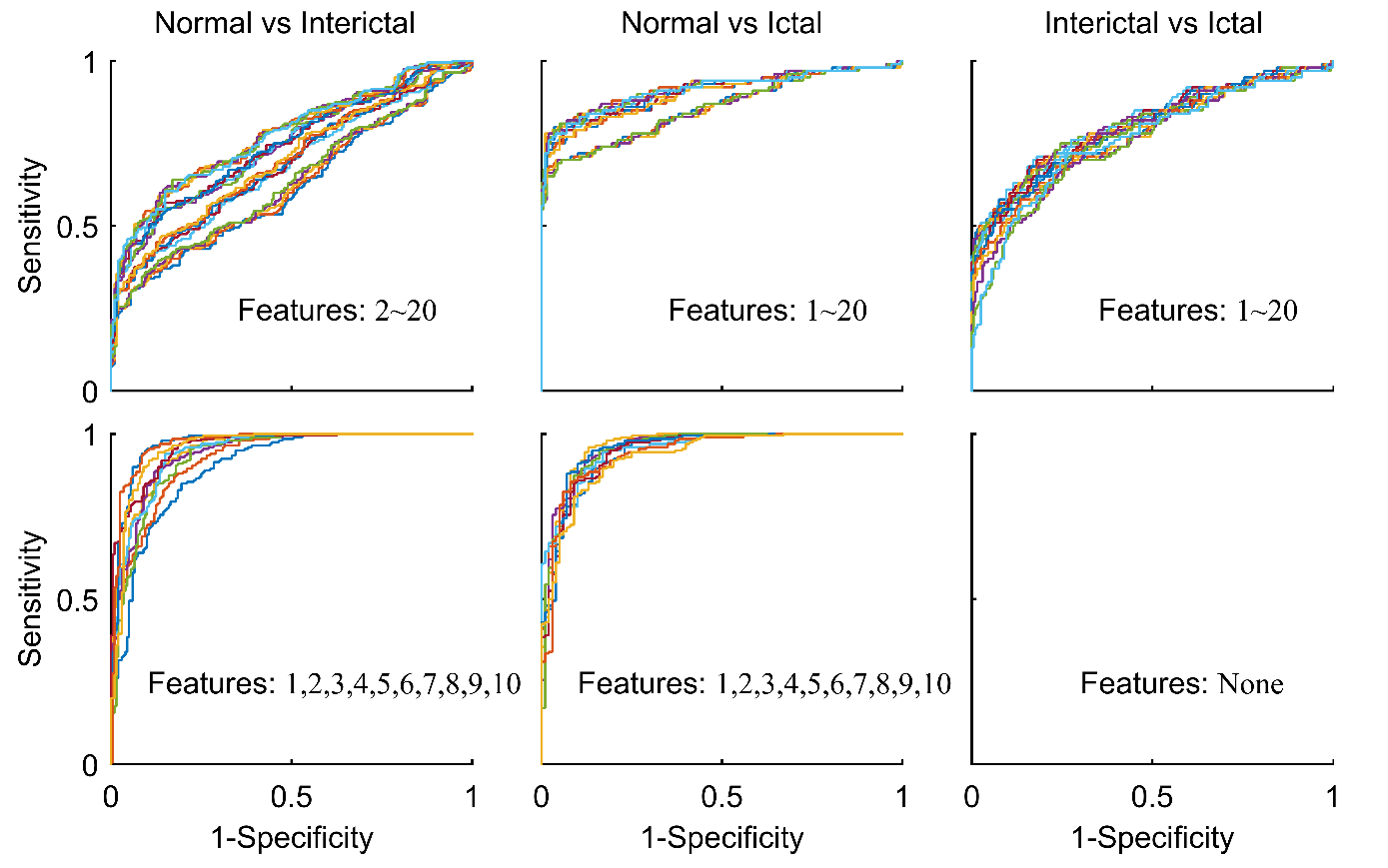


Supplementary Figure 1: ROC curves of DistEn (upper panel) and SampEn (lower panel) for detecting interictal EEG from normal (left panel), ictal EEG from normal (middle panel), and ictal EEG from interictal (right panel). Results correspond to the 5 s segment selection protocol A. Lines in different colors show different features with indices 1 to 20. Only several SampEn features are shown because the rest of them do not indicate a significant difference.


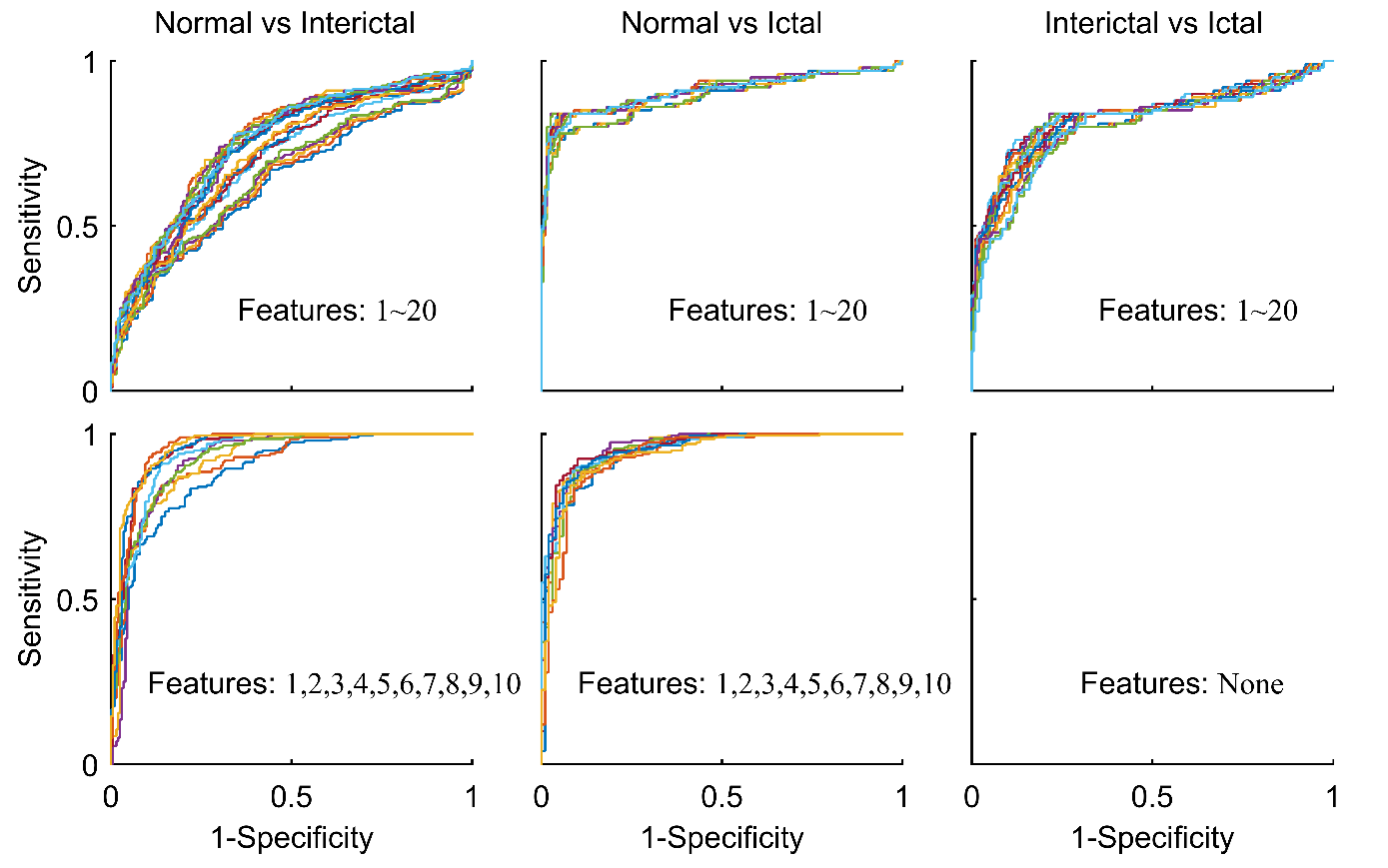


Supplementary Figure 2: ROC curves of DistEn (upper panel) and SampEn (lower panel) for detecting interictal EEG from normal (left panel), ictal EEG from normal (middle panel), and ictal EEG from interictal (right panel). Results correspond to the the 5 s segment selection protocol B. Lines in different colors show different features with indices 1 to 20. Only several SampEn features are shown because the rest of them do not indicate a significant difference.


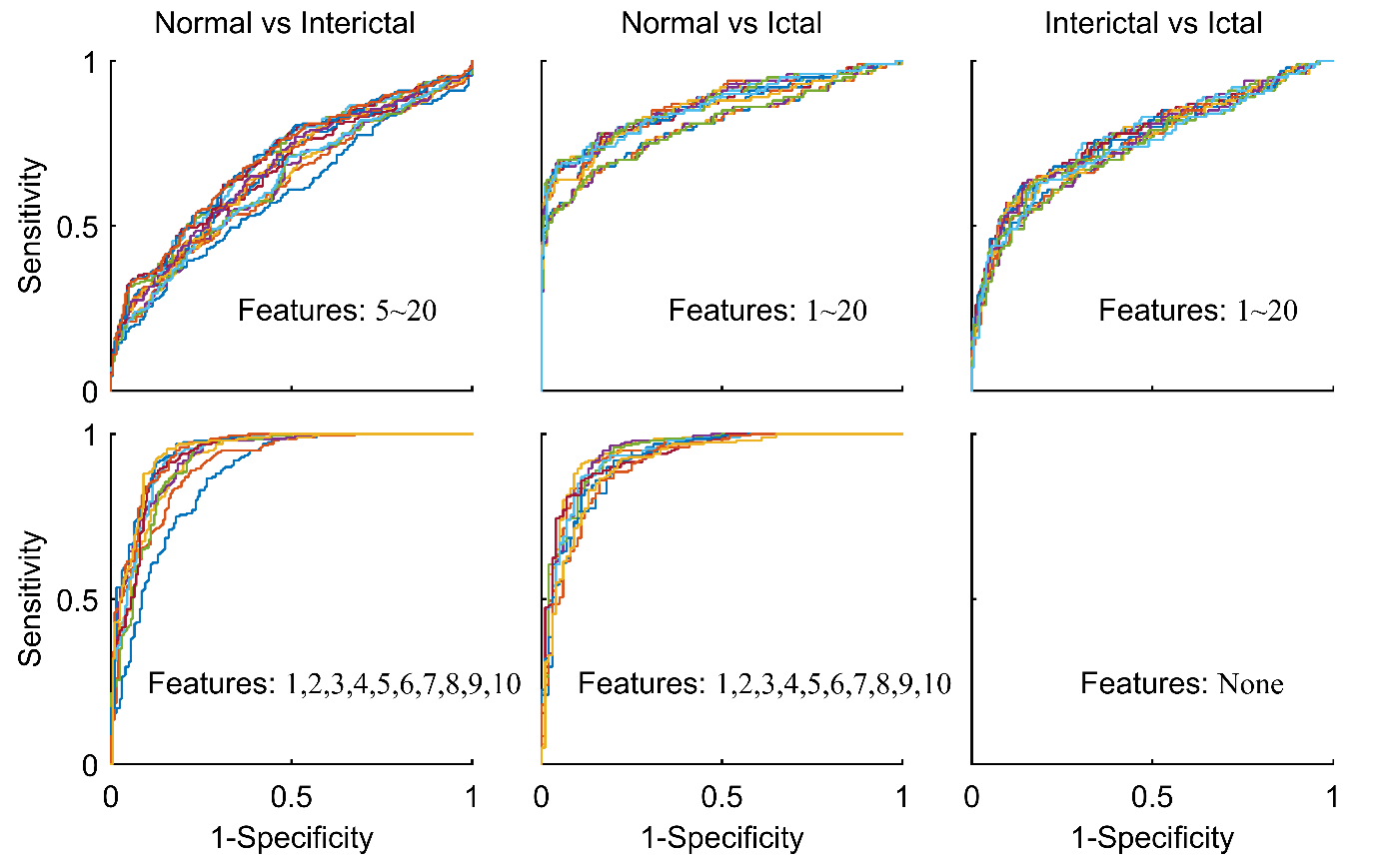


Supplementary Figure 3: ROC curves of DistEn (upper panel) and SampEn (lower panel) for detecting interictal EEG from normal (left panel), ictal EEG from normal (middle panel), and ictal EEG from interictal (right panel). Results correspond to the the 5 s segment selection protocol C. Lines in different colors show different features with indices 1 to 20. The first four DistEn features for detecting interictal from normal groups are not shown because no significant difference was found (see Table 1). Only several SampEn features are shown because the rest of them do not indicate significant difference.

# Algorithm of Determining $\left[ \boldsymbol{m,\tau} \right]$ for Distribution Entropy and Sample Entropy

The embedding dimension $m$ and time delay $\tau$ together determine whether the state space reconstruction of a time-series is appropriate or not. They both are important determinant parameters for DistEn and SampEn. However, in most studies including the reviewed EEG-related studies in this paper (Song et al., 2012; Acharya et al., 2015; Xiang et al., 2015), $m$ is usually set at $m=2$ and $\tau$ at $\tau=1$. This combination of $m$ and $\tau$ may be suitable for ‘discrete’ time-series such as heartbeat interval series (Grassberger et al., 1991; Pincus, 1991), but may not for ‘continuous’ time-series like EEG signals, since EEG recorded from different devices have different sampling frequencies varying from hundreds of to thousands of Hz. For a certain process, different sampling frequencies will lead to different oscillation features such as the auto-correlation time which will in turn affect the determination of $m$ and $\tau$ (Thuraisingham and Gottwald, 2006; Govindan et al., 2007). For example, if the temporal span of $m\cdot\tau$ is too small, the signal variation within the vector in the state space is mostly governed by noise. Similar to SampEn, DistEn was first developed without the introduction of the parameter $\tau$ because it was initially constructed for analysing heartbeat interval data (Li et al., 2015).

Several methods exist for determining the optimal values for $m$ and $\tau$ either separately (Fraser and Swinney, 1986; Kennel et al., 1992) or jointly (Gautama et al., 2003). In this study, we use a differential entropy based method to determine the optimal $m$ and $\tau$ jointly in order to avoid them falling into a non-optimal range (Gautama et al., 2003). We summarize this algorithm for a time-series of $N$ points $\left\{ u\left( i \right), 1\leq i\leq N \right\}$ as follows.

- Differential entropy estimation: Estimate the differential entropy of the time-series using the Kozachenko-Leonenko estimator $H\left( u,m,\tau\right)=\left[ \sum_{i=1}^{N} \ln\left( N\rho_{i}\left( m,\tau\right) \right) \right]/N+\ln2+C_{E}$, where $H\left( u \right)$ indicates the differential entropy of time-series $\left\{ u\left( i \right) \right\}$, $\rho_{i}\left( m,\tau\right)$ is the Euclidean distance of the $i$-th delay vector (in the state space representation of $\left\{ u\left( i \right) \right\}$ with the parameters $m$ and $\tau$) to its nearest neighbor, and $C_{E}\approx0.5772$ is the Euler constant (Beirlant et al., 1997).
- Ratio calculation: The ratio is defined as the ratio of the differential entropy of the time-series $\left\{ u\left( i \right) \right\}$ to the differential entropy of its surrogate time-series. So, we first generate $s$ surrogates using the iterated amplitude adjusted Fourier transform method (Schreiber and Schmitz, 2000). Then, the differential entropy of each surrogate is estimated in the same way as described in the former step. Define the ratio as $I\left( m,\tau\right)={H\left( u,m,\tau\right)}/{\left\langle H\left( \hat{u},m,\tau\right) \right\rangle_{s}}$, where $\hat{u}$ indicates the surrogate time-series and $\left\langle\cdot\right\rangle_{s}$ the average over $s$. In this study, we set $s$ at $s=5$ as has applied in (Gautama et al., 2003).
- Entropy ratio calculation: The entropy ratio is calculated by

$\mathbf{R}\left( \boldsymbol{m,\tau} \right)\boldsymbol{=}\mathbf{I}\left( \boldsymbol{m,\tau} \right)\left( \boldsymbol{1+}\frac{\boldsymbol{m}\ln\boldsymbol{N}}{\boldsymbol{N}} \right)$. (1)

The above algorithm needs firstly to define an initial sets for both $m$ and $\tau$ and those corresponding entropy ratios are obtained which yielding an $R$ map towards $m$ and $\tau$. The optimal combination of $m$ and $\tau$ occurs with the minimum of $R$. In this study, we searched for the optimal $m$ and $\tau$ values for each EEG segment with the initial sets of $m$ within [2, 10] and $\tau$ within [1, 15].

# Results for the Selection of $\boldsymbol{m}$ and $\boldsymbol{\tau}$

The minima of the entropy ratio $R$ maps did not always indicate the same combination of the optimal $m$ and $\tau$ for all the $500\times3$ EEG segments. However, we could observe some values that are frequently yielded, i.e., the optimal values for $m$ were focused on 2, 3, 4, and 5, and for $\tau$ on 8, 9, 10, 11, and 12. **Supplementary Figure** **1** shows the $R$ maps for five EEG segments from set Z, O, N, F, and S, respectively, with their minima indicating by open circles. In this study, we employed all the possible combinations of those $m$ and $\tau$ values, yielding thus a total of 20 DistEn/SampEn values for each EEG segment.


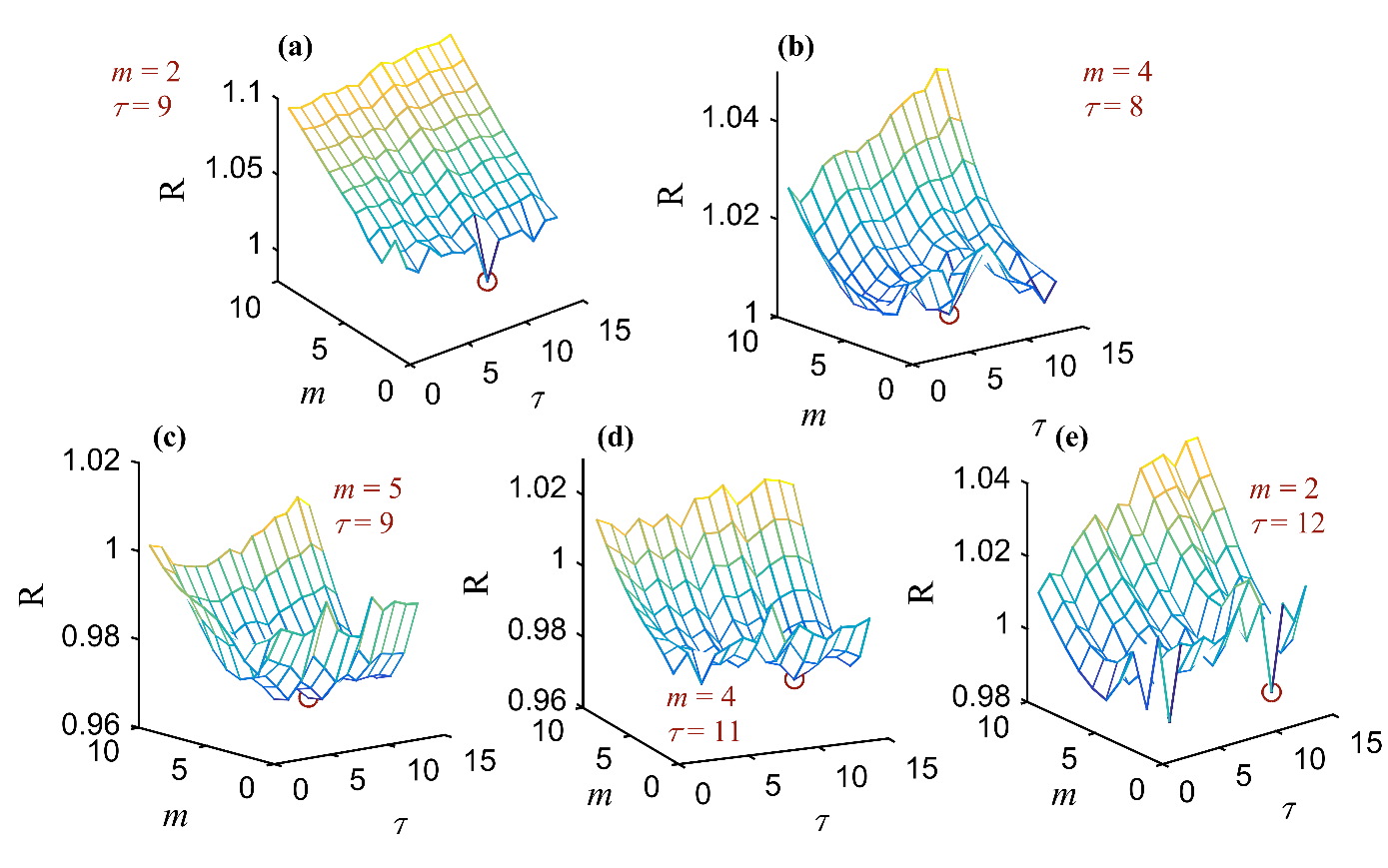


Supplementary Figure 4: Plots of the differential entropy ratio $\mathbf{R}$ for five exemplary EEG segments. From (a) to (e), EEG segment is randomly selected from set Z, O, N, F, and S, respectively. Minima of the plots are marked by open circles, which indicate the optimal values for $\boldsymbol{m}$ and $\boldsymbol{\tau}$.

# Source Code for DistEn

Following source code can be used to calculate DistEn value of a time-series on MATLAB platform.

| function DistEn = disten(sig, m, tau, B)  %DISTEN distribution entropy  %  % Input parameters  % ------------------------------------------------  % sig: Signal (time-series) under analysis  % m: Embedding dimension  % tau: Time delay  % B: Number of histogram bins  %  % Output parameters  % ------------------------------------------------  % DistEn: Distribution entropy value  %  % $Date: 17 Jun 2015  % $Modif.:  %    % parse inputs  narginchk(4, 4);    % rescaling  sig = (sig - min(sig)) ./ (max(sig) - min(sig));    % distance matrix  N = length(sig) - (m-1)*tau;  ind = hankel(1:N, N:length(sig));  rnt = sig(ind(:, 1:tau:end));  dv = pdist(rnt, 'chebychev');    % esimating probability density by histogram  num = hist(dv, linspace(0, 1, B));  freq = num./sum(num);    % disten calculation  DistEn = -sum(freq.*log2(freq+eps)) ./ log2(B); |
| --- |

# References

Acharya, U.R., Fujita, H., Sudarshan, V.K., Bhat, S., and Koh, J.E.W. (2015). Application of entropies for automated diagnosis of epilepsy using EEG signals: A review. *Knowledge-Based Systems* 88**,** 85-96. doi: 10.1016/j.knosys.2015.08.004.

Beirlant, J., Dudewicz, E.J., Györfi, L., and Van der Meulen, E.C. (1997). Nonparametric entropy estimation: An overview. *International Journal of Mathematical and Statistical Sciences* 6**,** 17-39.

Fraser, A.M., and Swinney, H.L. (1986). Independent coordinates for strange attractors from mutual information. *Physical Review A* 33**,** 1134-1140. doi: 10.1103/PhysRevA.33.1134.

Gautama, T., Mandic, D.P., and Van Hulle, M.M. (2003). "A differential entropy based method for determining the optimal embedding parameters of a signal", in: *Proceedings, 2003 IEEE International Conference on Acoustics, Speech, and Signal Processing*, 29.

Govindan, R.B., Wilson, J.D., Eswaran, H., Lowery, C.L., and Preißl, H. (2007). Revisiting sample entropy analysis. *Physica A: Statistical Mechanics and its Applications* 376**,** 158-164. doi: 10.1016/j.physa.2006.10.077.

Grassberger, P., Schreiber, T., and Schaffrath, C. (1991). Nonlinear time sequence analysis. *International Journal of Bifurcation and Chaos* 01**,** 521-547. doi: 10.1142/S0218127491000403.

Kennel, M.B., Brown, R., and Abarbanel, H.D. (1992). Determining embedding dimension for phase-space reconstruction using a geometrical construction. *Physical Review A* 45**,** 3403-3411. doi: 10.1103/PhysRevA.45.3403.

Li, P., Liu, C., Li, K., Zheng, D., Liu, C., and Hou, Y. (2015). Assessing the complexity of short-term heartbeat interval series by distribution entropy. *Medical & Biological Engineering & Computing* 53**,** 77-87. doi: 10.1007/s11517-014-1216-0.

Pincus, S.M. (1991). Approximate entropy as a measure of system complexity. *Proceedings of the National Academy of Sciences of the United States of America* 88**,** 2297-2301. doi: 10.1073/pnas.88.6.2297.

Schreiber, T., and Schmitz, A. (2000). Surrogate time series. *Physica D: Nonlinear Phenomena* 142**,** 346-382. doi: 10.1016/S0167-2789(00)00043-9.

Song, Y., Crowcroft, J., and Zhang, J. (2012). Automatic epileptic seizure detection in EEGs based on optimized sample entropy and extreme learning machine. *Journal of Neuroscience Methods* 210**,** 132-146. doi: 10.1016/j.jneumeth.2012.07.003.

Thuraisingham, R.A., and Gottwald, G.A. (2006). On multiscale entropy analysis for physiological data. *Physica A: Statistical Mechanics and its Applications* 366**,** 323-332. doi: 10.1016/j.physa.2005.10.008.

Xiang, J., Li, C., Li, H., Cao, R., Wang, B., Han, X., et al. (2015). The detection of epileptic seizure signals based on fuzzy entropy. *Journal of Neuroscience Methods* 243**,** 18-25. doi: 10.1016/j.jneumeth.2015.01.015.
