# Supplementary figures and images for: Classification of 5-S Epileptic EEG Recordings Using Distribution Entropy and Sample Entropy
Source: Front Physiol. 2016 Apr 14;7:136. doi: 10.3389/fphys.2016.00136 (PMC4830849; doi:10.3389/fphys.2016.00136)

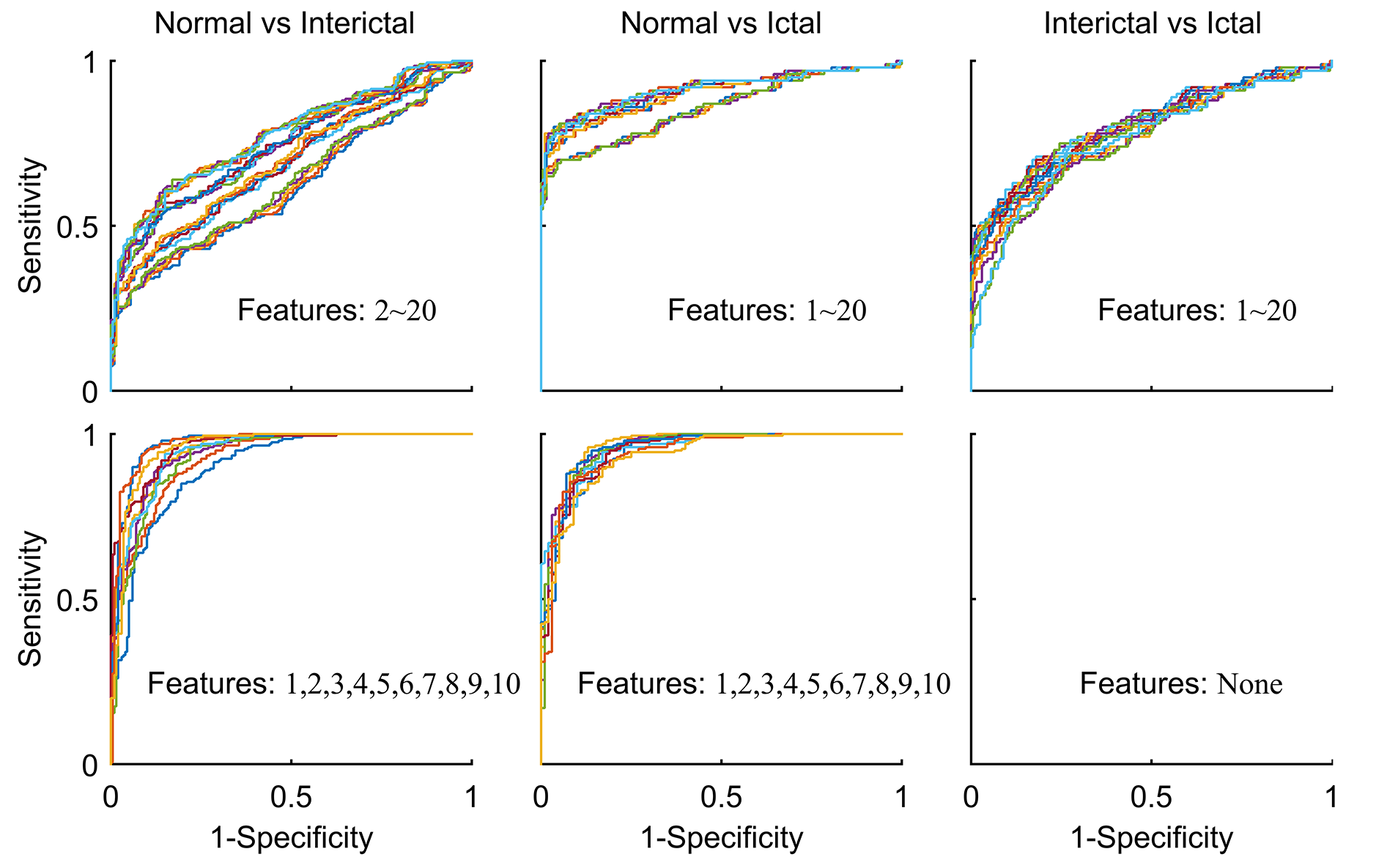

Supplement: Supplementary file 2 [file Image1.TIF]

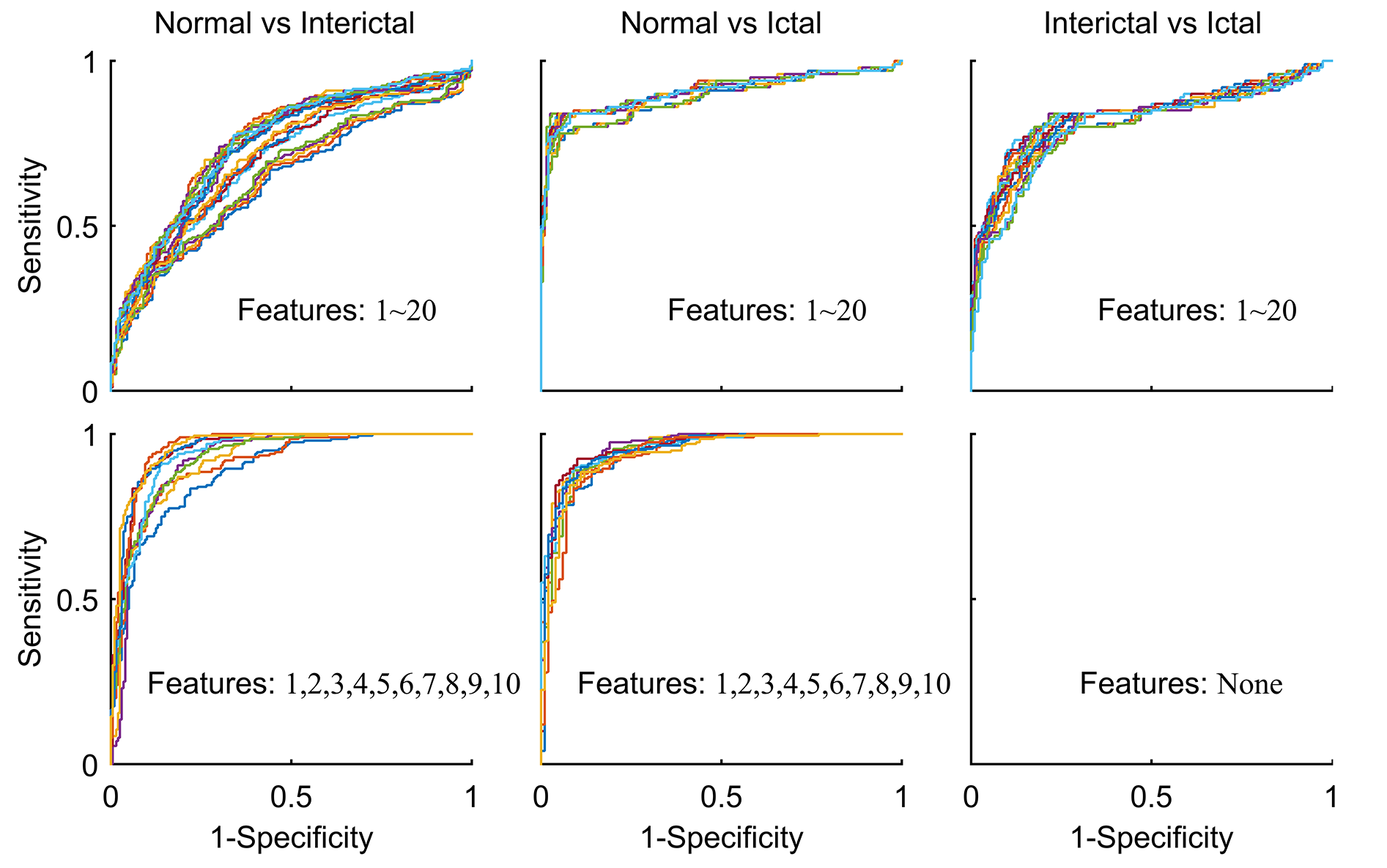

Supplement: Supplementary file 3 [file Image2.TIF]

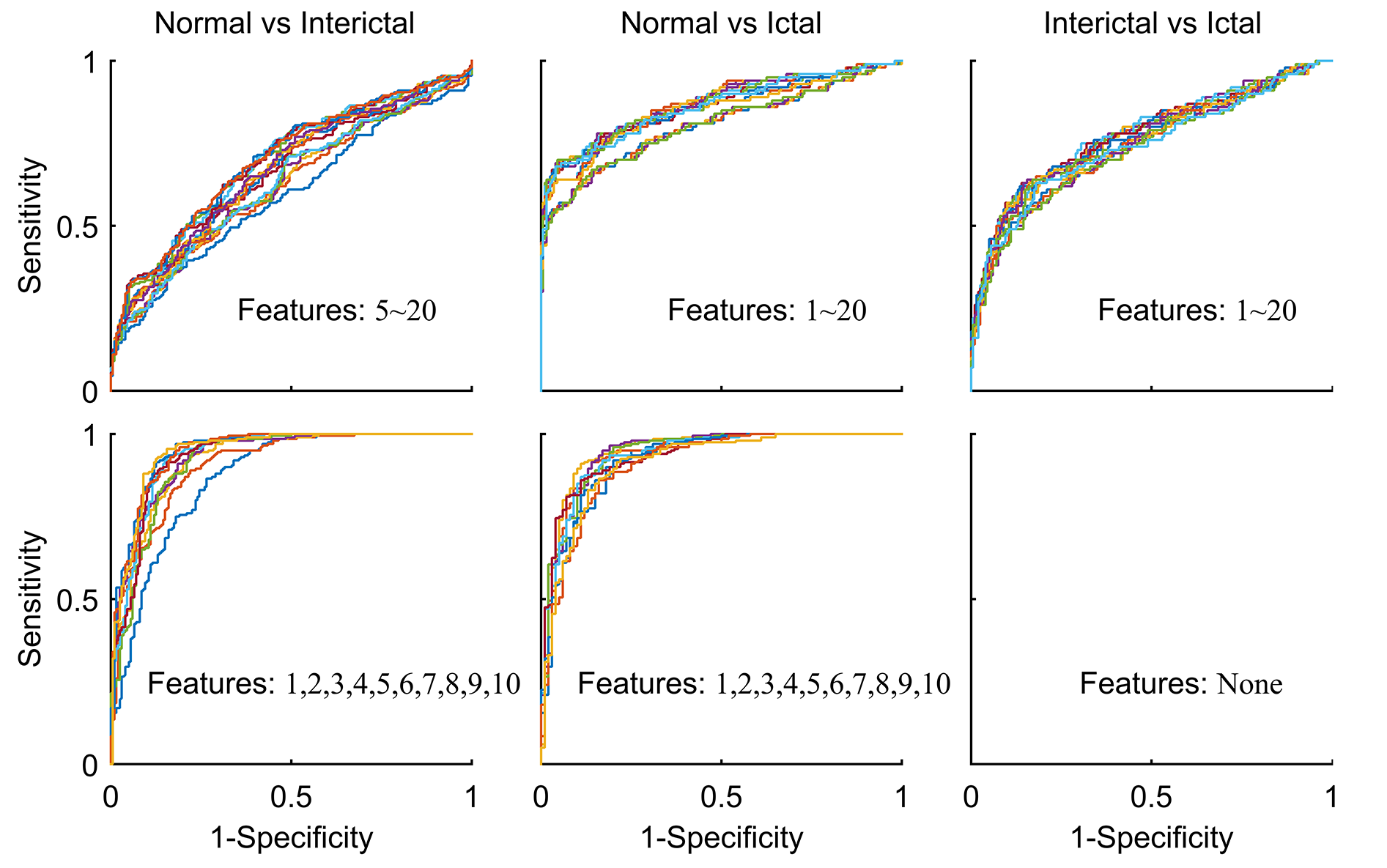

Supplement: Supplementary file 4 [file Image3.TIF]

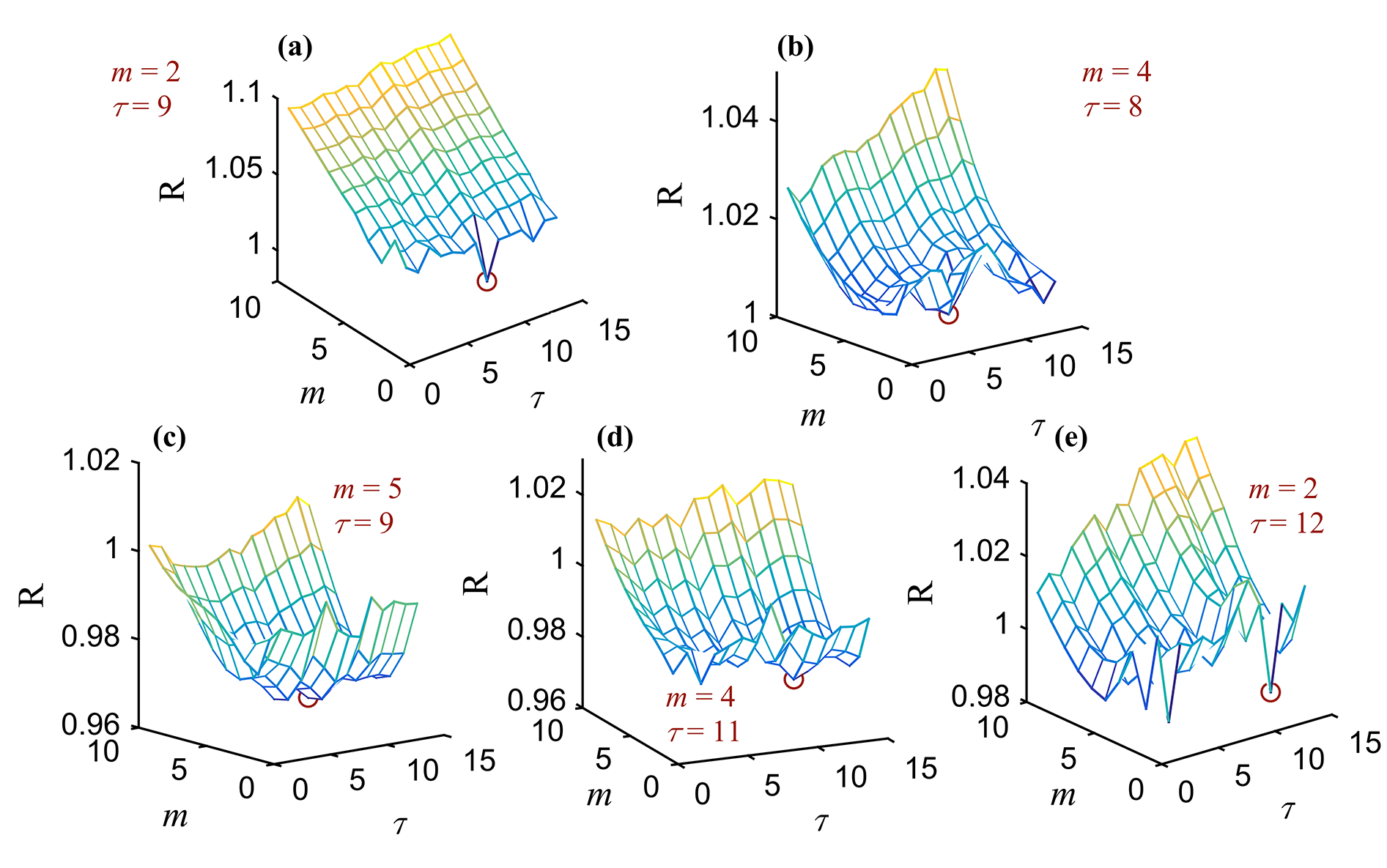

Supplement: Supplementary file 5 [file Image4.TIF]
